# Link between triglyceride-glucose-body mass index and future stroke risk in middle-aged and elderly chinese: a nationwide prospective cohort study

**DOI:** 10.1186/s12933-024-02165-7

**Published:** 2024-02-24

**Authors:** Yuankai Shao, Haofei Hu, Qiming Li, Changchun Cao, Dehong Liu, Yong Han

**Affiliations:** 1 Department of Emergency, Shenzhen Second People’s Hospital,The First Affiliated Hospital of Shenzhen University, No. 3002 Sungang Road, Futian District, Shenzhen, 518035 Guangdong China; 2 Department of Nephrology, Shenzhen Second People’s Hospital, Shenzhen, 518035 Guangdong China; 3 Department of Rehabilitation, Shenzhen Dapeng New District Nan’ao People’s Hospital, Shenzhen, 518000 Guangdong China

**Keywords:** Stroke, Triglyceride glucose-body mass index, Non-linear association, Insulin resistance, Smooth curve fitting

## Abstract

**Objective:**

Current literature is deficient in robust evidence delineating the correlation between the triglyceride glucose-body mass index (TyG-BMI) and the incidence of stroke. Consequently, this investigation seeks to elucidate the potential link between TyG-BMI and stroke risk in a cohort of middle-aged and senior Chinese individuals.

**Methods:**

This study employs longitudinal data from four waves of the China Health and Retirement Longitudinal Study (CHARLS) conducted in 2011, 2013, 2015, and 2018, encompassing 8,698 participants. The CHARLS cohort was assembled using a multistage probability sampling technique. Participants underwent comprehensive evaluations through standardized questionnaires administered via face-to-face interviews. Our analytic strategy involved the application of Cox proportional hazards regression models to investigate the association between TyG-BMI and the risk of stroke. To discern potential non-linear relationships, we incorporated Cox proportional hazards regression with smooth curve fitting. Additionally, we executed a battery of sensitivity and subgroup analyses to validate the robustness of our findings.

**Results:**

Our study utilized a multivariate Cox proportional hazards regression model and found a significant correlation between the TyG-BMI and the risk of stroke. Specifically, a 10-unit increase in TyG-BMI corresponded to a 4.9% heightened risk of stroke (HR = 1.049, 95% CI 1.029–1.069). The analysis also uncovered a non-linear pattern in this relationship, pinpointed by an inflection point at a TyG-BMI value of 174.63. To the left of this inflection point—meaning at lower TyG-BMI values—a 10-unit hike in TyG-BMI was linked to a more substantial 14.4% rise in stroke risk (HR 1.144; 95% CI 1.044–1.253). Conversely, to the right of the inflection point—at higher TyG-BMI values—each 10-unit increment was associated with a smaller, 3.8% increase in the risk of stroke (HR 1.038; 95% CI 1.016–1.061).

**Conclusions:**

In the middle-aged and elderly Chinese population, elevated TyG-BMI was significantly and positively associated with stroke risk. In addition, there was also a specific non-linear association between TyG-BMI and stroke (inflection point 174.63). Further reduction of TyG-BMI below 174.63 through lifestyle changes and dietary control can significantly reduce the risk of stroke.

**Supplementary Information:**

The online version contains supplementary material available at 10.1186/s12933-024-02165-7.

## Introduction

A stroke is medically characterized as an acute episode of neurological dysfunction, typically resulting from either a hemorrhage or an obstruction in blood flow, with symptoms persisting for more than 24 h or leading to death [1]. As a major health concern, stroke is associated with high rates of death, long-term disability, and limited effective treatments [2–4]. Data from the 2019 Global Burden of Disease study reveal that strokes have escalated from being the fifth to the third leading contributor to the global health burden since 1990 [5]. Projections indicate that, in the absence of effective preventive measures, annual stroke fatalities could reach between 7 and 8 million by the year 2030 [6]. The financial burden on families and society is enormous. Thus, the identification and management of stroke risk factors are crucial for prevention and for alleviating societal financial strains.

There is growing evidence that insulin resistance (IR) is recognized as a new risk factor for stroke and an early sign of type 2 diabetes, including not only diabetic but also nondiabetic individuals [7–10]. There are several methods to assess IR, and the Homeostasis Model Assessment of IR (HOMA-IR) has been widely used and shown its effectiveness in predicting cardiovascular disease [11]. Nevertheless, its requirement for fasting insulin levels limits its practicality in clinical settings. Studies have confirmed that the triglyceride-glycemic index (TyG), which consists of the product of fasting plasma glucose (FPG) levels and triglycerides (TG), is a simple, reproducible, and reliable index for assessing IR [12–14]. Many studies have confirmed the association of this index with stroke risk [15–18]. In addition, a study found that TyG is superior to HOMA-IR in predicting stroke risk [19]. Recently, there has been a surge of interest in a metric known as the triglyceride glucose-body mass index (TyG-BMI), which is the product of body mass index (BMI) and the TyG index. The TyG-BMI captures multiple clinical variables, such as BMI, glycemia, and lipid profiles simultaneously, and is more reflective of IR than the individual indices [20]. Studies have demonstrated that TyG-BMI is significantly associated with diabetes, hypertension, and nonalcoholic fatty liver disease (NAFLD) [21–23].

Since there is a significant association between IR and stroke, we hypothesized that TyG-BMI may be a valid predictor of stroke. Unfortunately, studies on the association between TyG-BMI and stroke are very limited, with only one cross-sectional study addressing this topic [24]. Besides, there are no studies investigating the non-linear relationship between them. In addition, studies differed in terms of implementation time, TyG-BMI range, sex ratio, and adjustment factors. Therefore, the relationship between TyG-BMI and stroke risk in the Chinese population remains unclear. To test this hypothesis, we embarked on a prospective cohort study using data from the China Health and Retirement Longitudinal Study (CHARLS) 2011–2018.

## Methods

### Study design

This cohort study used data from CHARLS from 2011 to 2018. TyG-BMI was considered the primary independent variable, and the incidence of stroke, coded as a binary variable (stroke = 1, no stroke = 0), served as the outcome of interest.

### Data sources and study population

The data for this investigation were sourced from the China Health and Retirement Longitudinal Study (CHARLS), a comprehensive national cohort study designed to evaluate the economic, social, and health circumstances of the population [25]. The CHARLS cohort was established through a multistage probability sampling process, selecting participants from 450 communities across 150 counties in 28 provinces, resulting in 10,257 households included in the initial survey. The baseline survey included targeted individuals aged 45 and older as of the survey period, which spanned from June 2011 to March 2012. Data were collected via standardized questionnaires administered through personal interviews, with follow-up interviews conducted biennially. The study received ethical approval from the Biomedical Ethics Review Board of Peking University in China (IRB00001052-11015). All study participants provided written consent prior to their inclusion. The dataset and Additional file 1 pertinent to this study are publicly accessible on the CHARLS project’s website [25].

**Fig. 1:**
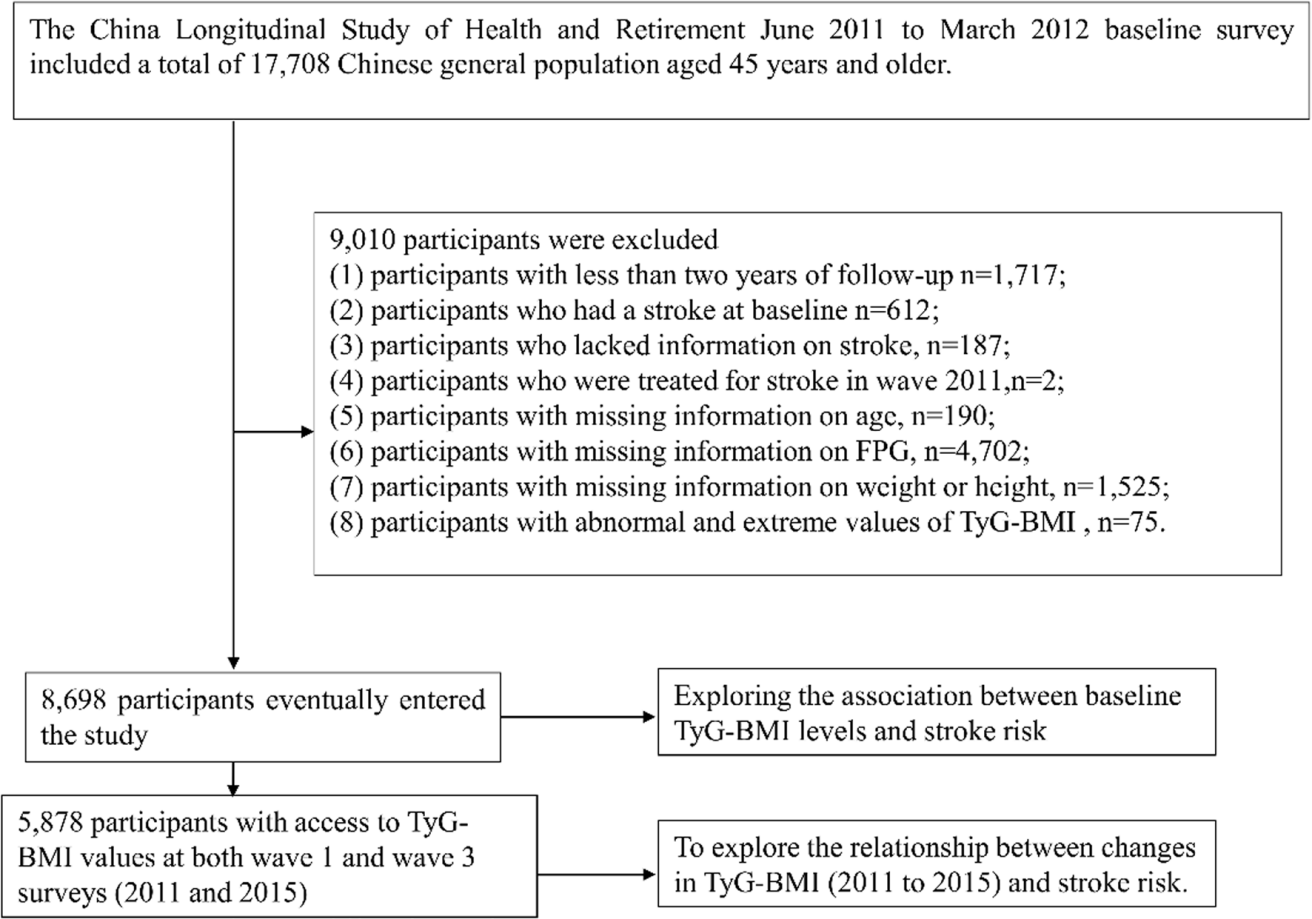
Flowchart illustrating the selection process of study participants

Our investigation drew upon data from waves of the CHARLS survey conducted in the years 2011, 2013, 2015, and 2018 [25]. The initial 2011–2012 baseline survey included 17,708 respondents. To refine our study group, we applied several exclusion criteria. Initially, we removed 1717 individuals who had been followed for less than two years. We also omitted 612 participants who had experienced a stroke prior to the baseline survey, 187 individuals for whom stroke data were incomplete, and 2 subjects who had received stroke treatment during the 2011 survey phase. Further exclusions were made for 4702 participants lacking blood glucose measurements and 1525 without recorded height or weight data. We also excluded 75 cases with TyG-BMI values exceeding three standard deviations from the mean. After applying these criteria, our final analysis encompassed 8698 participants. In addition, to further explore the relationship between changes in TyG-BMI (2011 to 2015) and stroke risk. We further included 5878 participants who had access to TyG-BMI values at both wave 1 and wave 3 surveys. The detailed methodology of our participant selection process is depicted in Fig. 1.

## Variables

### Calculation of triglyceride glucose-body mass index

The specific procedure for defining TyG-BMI in this study was as follows: TyG-BMI was calculated as TyG-BMI = BMI × TyG index, where TyG index = ln [FPG (mg/dL) × TG (mg/dL)/2], and BMI = weight/height^2^ (kg/m^2^) [14, 20].

### Stroke diagnosis

Participants free of stroke at baseline who reported a stroke at subsequent follow-up were recorded as incident cases. Data on stroke occurrence were systematically gathered via a questionnaire inquiring if participants had been diagnosed with a stroke by a physician, the date of diagnosis or awareness of the condition, and whether they were undergoing treatment for their stroke [25, 26]. Affirmative responses during follow-up led to classification as first-time stroke diagnoses, with the reported date marking the onset. The interval between the stroke onset and baseline assessment was calculated to establish the timing of the stroke. For those without reported strokes during follow-up, we determined follow-up duration by the interval between the baseline assessment and their final survey date [25].

### Covariates

Covariates were chosen based on prior studies and clinical expertise [26, 27]. The following covariates were included: (i) categorical variables: smoking status, sex, chronic kidney disease(CKD), chronic lung disease(CLD), malignant tumors, diabetes mellitus (DM), drinking status, hypertension; (ii) continuous variables: age, serum high-density lipoprotein cholesterol (HDL-c), BMI, Cystatin C, diastolic blood pressure(DBP), total serum cholesterol (TC), hemoglobin concentration (HGB), hemoglobin A1c (HBA1c), platelet (PLT), serum triglyceride (TG), fasting plasma glucose (FPG), serum low-density lipoproteins cholesterol (LDL-c), systolic blood pressure (SBP), blood urea nitrogen (BUN), Uric acid(UA), serum creatinine (Scr), Estimated glomerular filtration rate (eGFR).

### Data collection

Interviewers trained by CHARLS staff at Peking University conducted in-home surveys using computer-assisted personal interviewing (CAPI) methods [25]. The core questionnaire of CHARLS included sections on demographics, health status, functioning, diagnosed chronic conditions, and health-related behaviors such as smoking, drinking, and exercise. These interviewers were also equipped to measure participants' physical health metrics, including height, weight, and blood pressure.

Participants were further requested to visit their nearest township hospital or local Centers for Disease Control and Prevention for comprehensive health evaluations. At these locations, trained nurses obtained an 8 mL fasting blood sample from each respondent. These samples underwent a complete blood count within one to two hours of collection. The samples were then prepared by separating plasma and red blood cells, followed by storage at − 20 °C for safe transportation. Finally, all collected blood samples were sent to Beijing for detailed analyses at the Chinese Center for Disease Control and Prevention [25].

To calculate the eGFR for “Asian origin” patients, the Chronic Kidney Disease Epidemiology Collaboration (CKD-EPI) formula was employed [28]. This estimation takes into account variables such as sex, age, and Scr levels. The eGFR for female patients with Scr levels at or below 0.7 mg/dL is determined by the equation eGFR = 151 × (Scr/0.7)^−0.328^ × (0.993)^age^. For female patients with Scr levels above 0.7 mg/dL, the eGFR is calculated as eGFR = 151 × (Scr/0.7)^−1.210^ × (0.993)^age^. In male patients with Scr levels at or below 0.9 mg/dL, the eGFR is calculated using eGFR = 149 × (Scr/0.9)^−0.415^ × (0.993)^age^, and for those with Scr levels above 0.9 mg/dL, the eGFR formula is eGFR = 149 × (Scr/0.9)^−1.210^ × (0.993)^age^. The unit of age and Scr was year and mg/dL, respectively.

### Missing data processing

In our study, there were missing data on BUN (1, 0.01%), smoking status (1, 0.01%), Scr (2, 0.02%), HDL-c (2, 0.02%), alcohol consumption (4, 0.05%), LDL-c (13, 0.15%), CLD (30, 0.34%), CKD (41, 0.47%), hypertension (46, 0.53%), DBP (61, 0.70%), HBA1c (62, 0.71%), DM (82, 0.94%), SBP (93, 1.06%), PLT (165, 1.90%), HGB (166, 1.91%), WBC (169, 1.94%), cystatin C (2090, 24.03%), and eGFR (2, 0.02%). In order to reduce bias due to missing variables, which prevented the modeling phase from accurately describing the statistical efficacy of the target sample, multiple imputations based on approaches reported by White and Groenwald for missing data [29, 30]. Age, LDL-c, drinking status, FPG, BUN, smoking status, HDL-c, CLD, eGFR, UA, malignancy, sex, TC, CKD, HBA1c, DM, PLT, and HGB were included in the estimation model (the number of iterations was 10, and the regression type was linear regression). The missing data analysis process used the missing at random (MAR) assumption [29].

### Statistical analyses

Statistical analyses were conducted using R language software version 3.4.3 and Empower(R) software version 4.0. Statistical significance was defined as P values below 0.05 (two-sided). Baseline indicators were categorized based on the quartiles of TyG-BMI, and a comparison of the baseline characteristics was made for individuals in each group. Continuous variables were presented as median (interquartile range) or mean (SD: standard deviation), while categorical variables were described using percentages and frequencies. Differences between TyG-BMI groups were tested using the χ^2^ test, and differences in continuous variables were analyzed using analysis of variance (ANOVA) and the Kruskal–Wallis H test.

Univariate and multivariate Cox regression analyses were employed to evaluate the relationships between TG, FPG, TyG, BMI, and TyG-BMI with the risk of stroke. Three models were used: Model I (not adjusted for any covariates), Model II (adjusted for sex and age), Model III (adjusted for age, CRP, eGFR, sex; HDL-c, LDL-c, UA, CLD, PLT, Cystatin C; hypertension, HBA1C, diabetes; CKD, smoking, and drinking status variables). The TC was excluded from the final multivariate Cox proportional hazards model due to collinearity with other predictors, as detailed in Additional file 1: Table S1.

Besides, an unsupervised machine learning technique, the K-means algorithm with Euclidean distance, was utilized to group patients based on their TyG-BMI measurements in 2011 and 2015 [31]. Subsequently, the relationship between changes in TyG-BMI and the risk of stroke was investigated using a multivariate logistic regression model. Previous studies have shown a significant association between diabetes, obesity, CKD and stroke [32–34]. Several sensitivity analyses were performed to validate the findings. First, participants without diabetes (n = 8224) were analyzed. In addition, participants with a BMI ≥ 24 kg/m^2^ were excluded from the sensitivity analyses (n = 5208) [35]. Besides, the association between TyG-BMI and stroke risk in participants without CKD (n = 8120) was explored. In addition, continuous covariates were included in the equations using generalized additive modeling (GAM). The E-value was calculated to assess the possibility of unmeasured confounders between TyG-BMI and stroke risk [36].

To explore potential non-linear associations between the TyG-BMI index and stroke risk, we employed Cox proportional hazards models with smooth curve fitting. Where nonlinearity emerged, a recursive algorithm pinpointed the inflection point. We then formulated a piecewise Cox proportional hazards model on either side of these inflection points. The optimal model describing the TyG-BMI-stroke risk relationship was determined through a log-likelihood ratio test.

Subgroup analyses of different subgroups (age, sex, hypertension, smoking status, and alcohol consumption status) were performed using stratified Cox proportional hazards regression models. In addition to stratification factors, we adjusted for age, CRP, eGFR, sex, HDL-c, LDL-c, UA, CLD, PLT, Cystatin C, hypertension, HBA1C, DM, CKD, smoking, and drinking status. To assess the presence of an interaction term, we used likelihood ratio tests in models with and without an interaction term.

## Results

### Participant characteristics

**Fig. 2:**
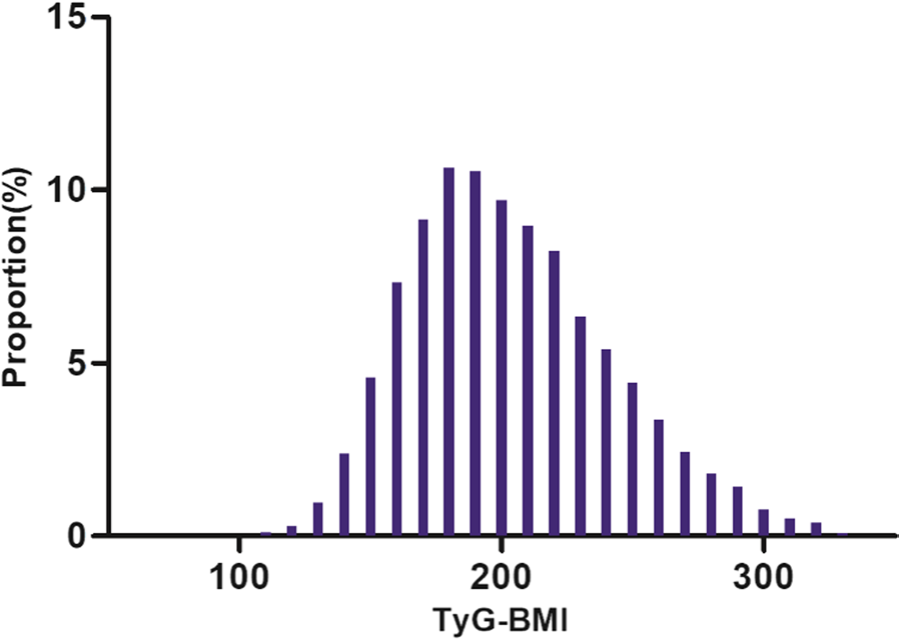
The distribution of TyG-BMI across the study population, indicating a normal distribution with a mean value of 203.52 kg/m^2^

A total of 8,698 participants, 4,008 males and 4,690 females, with a mean age of 59.36 (9.27) years, participated in the analysis. TyG-BMI was normally distributed, ranging from 88.45 to 331.39, with a mean (standard deviation) of 203.52 (38.66) (Fig. 2). Anthropometric and biochemical characteristics of patients stratified according to TyG-BMI quartiles are presented in Table 1. The results showed that various parameters such as FPG, BMI, TC, DBP, SBP, HBA1C, BMI, TyG, TG, UA, and HGB increased significantly with increasing TyG-BMI values. In contrast, age, HDL-c, BUN, and Scr showed opposite trends. In addition, the proportion of non-smokers, females, hypertensive disorders, and DM gradually increased with increasing TyG-BMI, whereas the proportion of males, CLD, and CKD gradually decreased.

**Table 1:** The baseline characteristics of participants

TyG-BMI quartile	Q1 (< 175.14)	Q2 (175.14–198.79)	Q3 (198.79–228.35)	Q4 (≥ 228.35)	P-value
Participants (n)	2175	2174	2174	2175	
Age (years, mean ± SD)	61.9 ± 9.9	59.4 ± 9.2	58.7 ± 8.9	57.5 ± 8.5	< 0.001
PLT (10^9^/L, mean ± SD)	208.41 ± 76.22	210.43 ± 72.23	209.89 ± 70.63	217.45 ± 71.30	< 0.001
WBC (10^9^/L, mean ± SD)	6.05 ± 1.98	6.15 ± 1.85	6.21 ± 1.78	6.55 ± 1.90	< 0.001
BUN (mg/L, mean ± SD)	45.60 ± 14.04	44.44 ± 13.02	43.21 ± 11.76	42.99 ± 11.95	< 0.001
FPG (mg/L, mean ± SD)	99.17 ± 18.79	104.95 ± 26.84	109.82 ± 32.37	125.99 ± 52.62	< 0.001
Scr (mg/dL, mean ± SD)	0.78 ± 0.24	0.78 ± 0.30	0.78 ± 0.18	0.78 ± 0.20	0.707
eGFR (mL/min·1.73 m^2^, mean ± SD)	96.54 ± 15.47	97.29 ± 15.28	96.84 ± 15.58	96.72 ± 15.99	0.433
TC (mg/dL, mean ± SD)	183.83 ± 35.25	190.64 ± 37.15	196.32 ± 37.42	203.63 ± 40.65	< 0.001
TG (mg/dL,median, quartile)	73.46 (57.53–94.69)	92.93 (71.02–124.79)	116.82 (87.61–161.96)	165.49 (120.36–243.38)	< 0.001
TyG(mean ± SD)	8.20 ± 0.44	8.49 ± 0.46	8.77 ± 0.52	9.26 ± 0.68	< 0.001
TyG-BMI (mean ± SD)	158.34 ± 12.84	186.80 ± 6.86	212.56 ± 8.35	256.36 ± 22.38	< 0.001
HDL-c (mg/dL, mean ± SD)	59.89 ± 15.79	54.75 ± 14.38	49.01 ± 13.04	42.16 ± 11.58	< 0.001
LDL-c (mg/Dl, mean ± SD)	109.75 ± 30.75	116.90 ± 32.86	120.86 ± 34.12	119.07 ± 40.05	< 0.001
CRP (mg/L median, quartile)	0.77 (0.44–1.81)	0.83 (0.48–1.80)	1.05 (0.58–2.07)	1.43 (0.80–2.73)	< 0.001
HBA1C (%, mean ± SD)	5.09 ± 0.52	5.18 ± 0.68	5.23 ± 0.70	5.56 ± 1.10	< 0.001
UA (mg/dL, mean ± SD)	4.29 ± 1.21	4.31 ± 1.19	4.49 ± 1.27	4.66 ± 1.27	< 0.001
HGB (g/L, mean ± SD)	13.96 ± 2.23	14.26 ± 2.26	14.31 ± 2.06	14.67 ± 2.28	< 0.001
SBP (mmHg, mean ± SD)	124.88 ± 21.75	126.96 ± 20.48	130.60 ± 21.21	134.43 ± 21.47	< 0.001
DBP (mmHg, mean ± SD)	71.81 ± 11.75	73.99 ± 11.72	76.57 ± 11.75	79.51 ± 12.29	< 0.001
Cystatin C (mg/L, mean ± SD)	1.06 ± 0.27	1.02 ± 0.32	0.99 ± 0.24	0.96 ± 0.25	< 0.001
BMI (kg/m^2^, mean ± SD)	19.36 ± 1.70	22.07 ± 1.34	24.30 ± 1.54	27.77 ± 2.51	< 0.001
Sex (N, %)					< 0.001
Male	1240 (57.01%)	1064 (48.94%)	912 (41.95%)	791 (36.37%)	
Female	935 (42.99%)	1110 (51.06%)	1262 (58.05%)	1384 (63.63%)	
Hypertension (N, %)	272 (12.51%)	362 (16.65%)	550 (25.30%)	872 (40.09%)	< 0.001
DM (N, %)	38 (1.75%)	68 (3.13%)	121 (5.57%)	247 (11.36%)	< 0.001
Malignant tumors (N, %)	25 (1.15%)	15 (0.69%)	18 (0.83%)	32 (1.47%)	0.051
CLD (N, %)	318 (14.62%)	220 (10.12%)	185 (8.51%)	194 (8.92%)	< 0.001
CKD (N, %)	170 (7.82%)	144 (6.62%)	139 (6.39%)	125 (5.75%)	0.049
Smoking status (N, %)					< 0.001
Never	1087 (49.98%)	1276 (58.69%)	1435 (66.01%)	1524 (70.07%)	
Ever	171 (7.86%)	167 (7.68%)	197 (9.06%)	208 (9.56%)	
Current	917 (42.16%)	731 (33.62%)	542 (24.93%)	443 (20.37%)	
Drinking status (N, %)					< 0.001
Never	311 (14.30%)	296 (13.62%)	300 (13.80%)	281 (12.92%)	
Ever	1226 (56.37%)	1281 (58.92%)	1357 (62.42%)	1463 (67.26%)	
Current	638 (29.33%)	597 (27.46%)	517 (23.78%)	431 (19.82%)	

*SD* standard deviation, *N* number, *LDL-c* low-density lipoproteins cholesterol, *BUN* blood urea nitrogen, *TyG-BMI* Triglyceride glucose-body mass index, *WBC* white blood cell count, *PLT* platelet, *BMI* body mass index, *HGB* hemoglobin concentration, *UA* Uric acid, *TC* total cholesterol, *HBA1c* hemoglobin A1c, *DBP* diastolic blood pressure, *TG* triglyceride, *eGFR* Estimated glomerular filtration rate, *CLD* Chronic Lung Diseases, *DM* diabetes mellitus, *Scr* serum creatinine, *HDL-c* high-density lipoprotein cholesterol, *CKD* Chronic kidney diseases, *SBP* systolic blood pressure

### The incidence rate of stroke

**Fig. 3:**
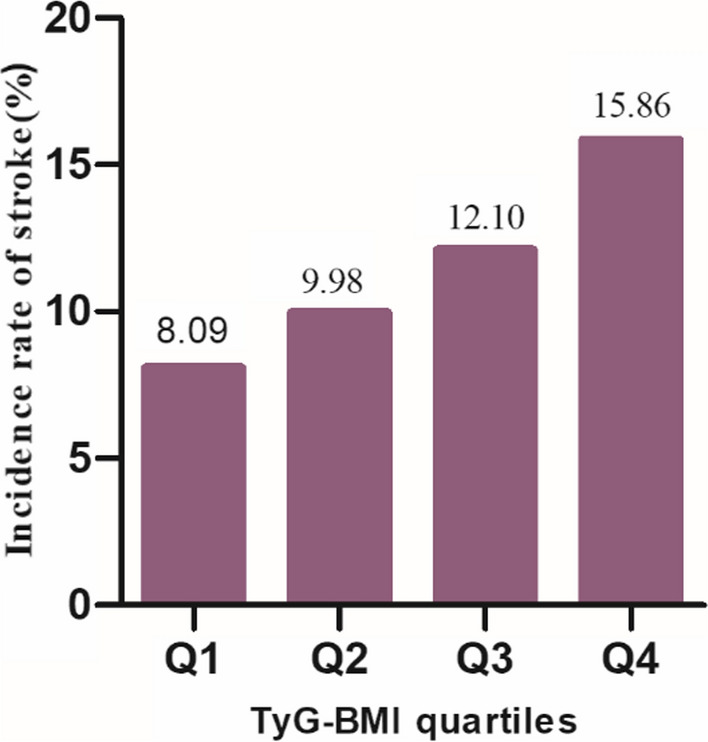
Bar chart represented the incidence of stroke across different quartiles of TyG-BMI

Table 2 showed that 1,001 participants had a stroke. The overall stroke incidence rate was 180 cases per 10,000 person-years. The stroke incidence rates for participants in the TyG-BMI quartiles were: Q1: 133.71/10000 person-years; Q2: 160.78/10000 person-years; Q3: 196.74/10000 person-years; Q4: 261.92/10000 person-years. The overall stroke incidence was 11.51%. The incidence for each TyG-BMI quartile was: Q1: 8.09%; Q2: 9.98%; Q3: 12.10%; Q4: 15.86% (Fig. 3). Participants with lower TyG-BMI had markedly lower stroke incidence compared to those with higher TyG-BMI.

**Table 2:** Incidence rate of stroke (% or Per 1000 person-year**)**

TyG-BMI	Participants (n)	Stroke events (n)	Incidence rate (95% CI) (%)	Per 10,000 person-year
Total	8698	1001	11.51(10.84–12.18)	187.92
Q1(< 175.14)	2175	176	8.09(6.95–9.24)	133.71
Q2 (175.14–198.79)	2174	217	9.98(8.72–11.24)	160.78
Q3 (198.79–228.35)	2174	263	12.10(10.73–13.47)	196.74
Q4 (≥ 228.35)	2175	345	15.86(14.33–17.40)	261.92
P for trend			< 0.001	

*TyG-BMI* triglyceride glucose-body mass index, *CI* confidence

**Fig. 4:**
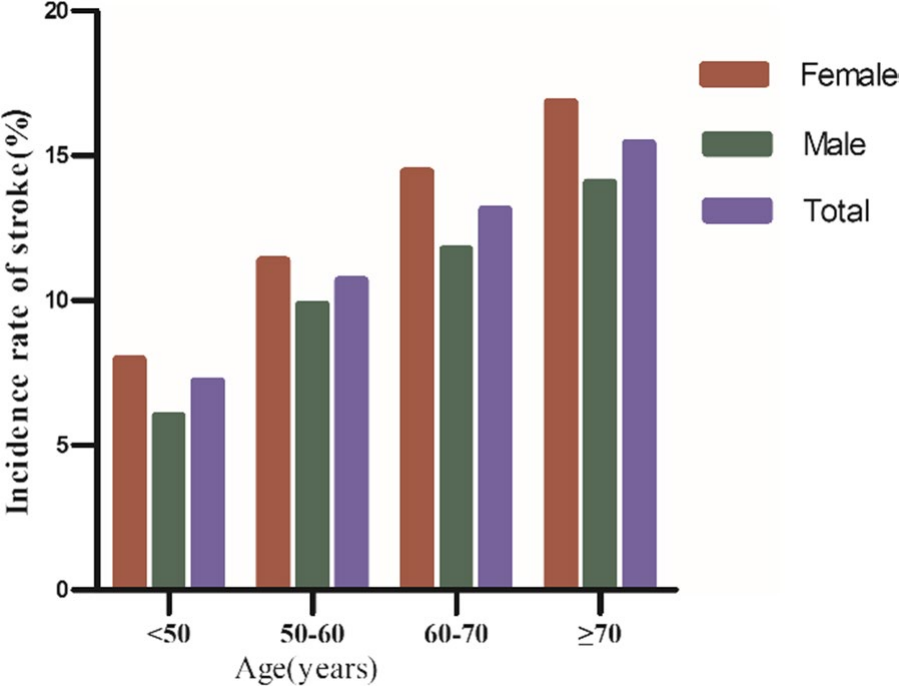
Comparative chart displayed the incidence of stroke across age groups, stratified by decade, highlighting that stroke incidence was higher in women than in men across all age groups and that stroke incidence increased with age for both genders

Regardless of age group, women had a greater incidence of stroke among individuals in the age stratification based on age < 50, 50 to < 60, 60 to < 70, and ≥ 70. Additionally, it was shown that incidence rose with age in both men and women (Fig. 4).

### Factors influencing the risk of stroke analyzed by univariate Cox proportional hazards regression

Based on univariate analyses, the risk of stroke was not related to BUN and HGB(P > 0.05), but was positively correlated with age, PLT, Scr, TC, TG, LDL-c, CRP, HBA1C, UA, Cystain C, FPG, hypertension, DM and current drinking, current smoking, whereas it was negatively associated with HDL-c and eGFR (all P < 0.05) (Additional fil 1: Table S2).

### Relationship between TyG-BMI and the risk of stroke

To explore the association between TyG-BMI and stroke risk, we developed three Cox proportional hazards regression models, detailed in Table 3. A 10-unit increment in TyG-BMI was associated with a 7.1% elevation in stroke risk in Model I (HR = 1.071, 95% CI 1.055–1.087). Model II demonstrated an 8.4% increase in stroke risk per 10-unit increase in TyG-BMI (HR = 1.084, 95% CI 1.067–1.101). In Model III, each 10-unit rise in TyG-BMI was linked to a 4.9% increase in the risk of stroke (HR = 1.049, 95% CI 1.029–1.069).

**Table 3:** Relationship between TyG-BMI and the risk of stroke in different models

	Model I (HR., 95%CI)	*P*	Model II (HR., 95%CI)	*P*	Model III (HR., 95%CI)	*P*	Model IV (HR., 95%CI)	*P*
TyG-BMI (per 10 units)	1.071 (1.055, 1.087)	< 0.001	1.084 (1.067, 1.101)	< 0.001	1.049 (1.029, 1.069)	< 0.001	1.046 (1.026, 1.067)	< 0.001
TyG-BMI quartile								
Q1	Ref		Ref		Ref		Ref	
Q2	1.197 (0.982, 1.461)	0.076	1.290 (1.057, 1.576)	0.012	1.238 (1.011, 1.517)	0.039	1.229 (1.003, 1.508)	0.047
Q3	1.461 (1.207, 1.768)	< 0.001	1.614 (1.330, 1.958)	< 0.001	1.387 (1.128, 1.706)	0.002	1.366 (1.109, 1.684)	0.003
Q4	1.945 (1.622, 2.332)	< 0.001	2.238 (1.858, 2.696)	< 0.001	1.611 (1.292, 2.010)	< 0.001	1.566 (1.249, 1.963)	< 0.001

Model I: we did not adjust other covariates

Model II: we adjust sex and age

Model III: we adjust age, CRP, eGFR, sex, HDL-c, LDL-c, UA, CLD, PLT, Cystatin C, hypertension, HBA1C, DM, CKD, smoking, and drinking status

Model IV: we adjust age(smooth), CRP (smooth), eGFR (smooth), sex; HDL-c(smooth), LDL-c(smooth), UA (smooth), CLD, PLT(smooth), Cystatin C(smooth), hypertension, HBA1C, diabetes; CKD, smoking, drinking status

*HR* hazard ratio, *Ref* reference, *CI* confidence

Besides, we further converted TyG-BM from a continuous variable to a categorical variable based on quartiles. The multivariable-adjusted model revealed that, with the lowest quartile (Q1) as the reference, the hazard ratios (HRs) for the subsequent quartiles (Q2, Q3, and Q4) in relation to stroke risk were as follows: Q2 had an HR of 1.238 (95% CI 1.011, 1.517), Q3 had an HR of 1.387 (95% CI 1.128, 1.706), and Q4 had an HR of 1.611 (95% CI 1.292, 2.010). This indicates that, compared to participants in Q1, those in Q2 had a 23.8% increased risk of stroke, those in Q3 had a 38.7% increased risk, and those in Q4 had a 61.1% increased risk (Table 3).

### Association of TG, FPG, TyG, and BMI with stroke risk based on multivariate Cox proportional hazards regression regression

The associations of TG, FPG, TyG, and BMI with stroke risk were further analyzed using multivariate Cox proportional hazards regression models (Additional file 1: Table S3). After adjusting for confounding variables, the results showed that there was no significant relationship between TG and FPG and stroke risk, with HR (95% CI, P) of 1.024 (95% CI 0.970–1.080, P = 0.389) and 1.015 (95% CI 0.987–1.044, P = 0.287), respectively. There was a significant positive relationship between BMI and the risk of stroke, with an increase in stroke risk of 4.5% for every 1 kg/m^2^ increase in BMI (HR = 1.045, 95% CI 1.025–1.064). In addition, TyG was significantly and positively associated with stroke risk, with each 1-unit increase in TyG increasing the risk of stroke by 16.5% (HR = 1.165, 95% CI 1.043–1.302).

### Sensitivity analysis

To ensure the integrity of our findings, sensitivity analyses were systematically conducted. Initially, TyG-BMI was categorized into quartiles and subsequently reincorporated into the regression model in its modified categorical form. It was observed that the intervals between effect sizes were uniform across the groups, and this pattern of effect sizes remained congruent with those observed when TyG-BMI was assessed as a continuous variable, as indicated in Table 3.

Additionally, we introduced the continuity covariate as a curve into the equation using a GAM. Table 3 illustrated that the results obtained from Model IV closely paralleled those from the fully adjusted model, exhibiting an HR of 1.046 with a 95% CI of 1.026–1.067, achieving statistical significance (P < 0.001).

Besides, participants were categorized into two groups based on their TyG-BMI measurements in 2011 and 2015 using a K-means with Euclidean distance. These included a group in which a change in TyG-BMI between 2011 and 2015 was observed, with overall low TyG-BMI values (Class 1), and a group in which a change was observed alongside high TyG-BMI values (Class 2). As depicted in Additional file 1: Fig. S1, it was observed that participants in Class 1 exhibited overall low TyG-BMI levels (2011: 182.77 ± 22.56; 2015: 185.26 ± 24.08), whereas overall high TyG-BMI levels were exhibited by participants in Class 2 (2011: 245.52 ± 27.03; 2015: 250.24 ± 31.61). Multifactorial logistic regression analysis showed a 24.1% increased stroke risk in participants of Class 2 compared to participants of Class 1 (odds ratio OR = 1.241, 95CI% 1.012–1.523, P = 0.038) (Additional file 1: Table S4).

Furthermore, the study conducted additional analyses to verify the strength and consistency of the relationship between TyG-BMI and the risk of stroke by focusing on specific groups and adjusting for various health factors (Table 4). Firstly, in a group without DM, consisting of 8,224 participants, the analysis adjusted for factors such as age, CRP, eGFR, sex, HDL-c, LDL-c, UA, CLD, PLT, Cystatin C, hypertension, HBA1C, CKD, smoking, drinking status. This analysis still showed a significant positive link between TyG-BMI and stroke risk (per 10 units, HR = 1.052, 95%CI 1.030–1.073, P < 0.001). Secondly, when participants with CKD were excluded, the results were similar after adjusting for confounding variables (including age, CRP, eGFR, sex; HDL-c, LDL-c, UA, CLD, PLT, Cystatin C; Hypertension, HBA1C, diabetes, smoking, drinking status), the TyG-BMI association with stroke risk remained positively significant, with an HR(95% CI) of 1.048 (1.027–1.070, per 10 units of TyG-BMI). Lastly, the analysis was narrowed down to participants with a BMI under 24 kg/m^2^, adjusting for all the previously mentioned factors (included age, CRP, eGFR, sex; HDL-c, LDL-c, UA, CLD, PLT, Cystatin C, CKD, hypertension, HBA1C, DM, smoking, drinking status.). The findings showed a significant positive association between TyG-BMI (per 10 units) and stroke risk, with an HR (95%CI) of 1.062 (1.016, 1.110). Furthermore, the E-value (1.28) was found to be greater than the relative risk of TyG-BMI and unmeasured confounders (1.26), suggesting that unknown or unmeasured variables may have little effect on the relationship between TyG-BMI and stroke risk. Based on all sensitivity analyses, our findings were robust.

**Table 4:** Relationship between TyG-BMI and the risk of stroke in different sensitivity analyses

	Model I (HR., 95%CI)	*P*	Model II (HR., 95%CI)	*P*	Model III (HR., 95%CI)	*P*
TyG-BMI (per 10 units)	1.052 (1.030, 1.073)	< 0.001	1.048 (1.027, 1.070)	< 0.001	1.062 (1.016, 1.110)	0.008
TyG-BMI quartiles						
Q1	Ref		Ref		Ref	
Q2	1.233 (1.002, 1.517)	0.047	1.243 (1.003, 1.540)	0.047	1.225 (0.992, 1.514)	0.059
Q3	1.384 (1.119, 1.713)	0.003	1.369 (1.101, 1.702)	0.005	1.311 (1.002, 1.716)	0.048
Q4	1.622 (1.289, 2.040)	< 0.001	1.598 (1.265, 2.018)	< 0.001	0.970 (0.508, 1.851)	0.926

Model I was a sensitivity analysis in participants without DM (n = 8224). Adjusted age, CRP, eGFR, sex; HDL-c, LDL-c, UA, CLD, PLT, Cystatin C, hypertension, HBA1C, DM, CKD, smoking, and drinking status

Model II was a sensitivity analysis conducted on non-CKD participants (n = 8120). Adjusted age, CRP, eGFR, sex; HDL-c, LDL-c, UA, CLD, PLT, Cystatin C, hypertension, HBA1C, DM, smoking, and drinking status

Model III was a sensitivity analysis conducted on participants with BMI < 24 kg/m^2^. (n = 5208). Adjusted age, CRP, eGFR, sex; HDL-c, LDL-c, UA, CLD, PLT, Cystatin C; hypertension, HBA1C, DM, CKD, smoking, drinking status

*HR* hazard ratio, *Ref* reference, *CI* confidence

### Cox proportional hazards regression model with smooth curve fitting to address nonlinearity

**Fig. 5:**
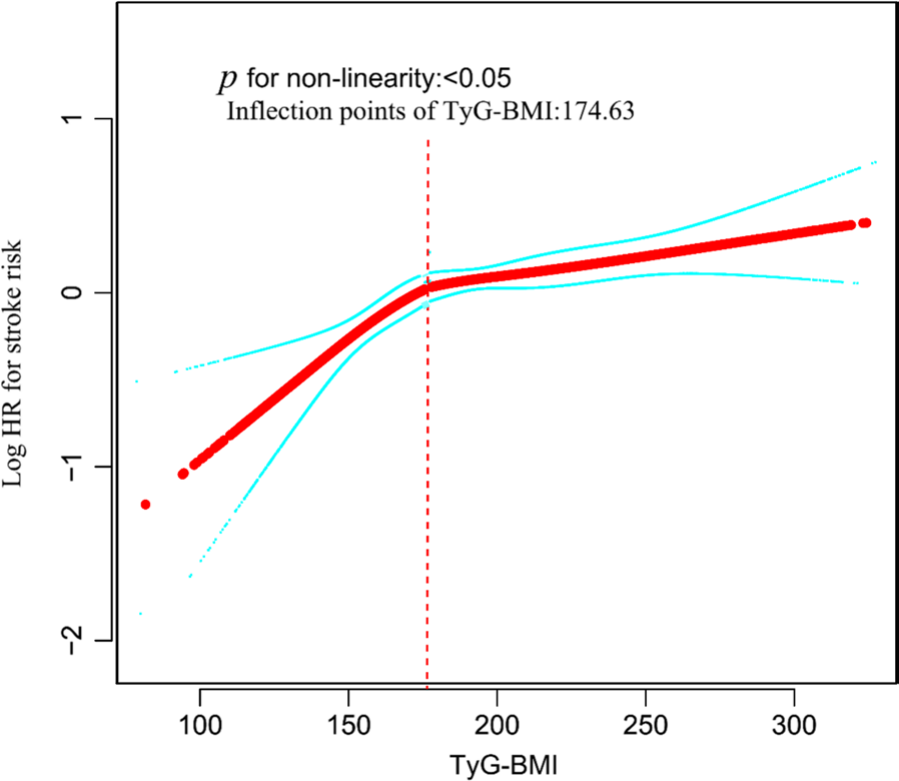
Curve plot demonstrated the non-linear relationship between TyG-BMI and stroke risk among all participants, adjusted for various factors including age, CRP, eGFR, sex, HDL-c, LDL-c, UA, CLD, PLT, Cystatin C, hypertension, HBA1C, DM, CKD, smoking, and drinking status

The nonlinearity of the association between TyG-BMI and stroke risk was discerned through the application of a Cox proportional hazards regression model with cubic spline functions, as depicted in Fig. 5. Subsequently, the most suitable model was ascertained via the log-likelihood ratio test, the results of which are detailed in Table 5, yielding a P-value of less than 0.05. An inflection point for the TyG-BMI was identified at 174.63 by employing a recursive algorithm. Post identification, a two-piecewise Cox proportional hazards regression model was utilized to ascertain the HRs and CIs on either side of this demarcation. It was observed that the HR stood at 1.144 (95% CI 1.044, 1.253) preceding the inflection point and 1.038 (95% CI 1.016–1.061) subsequent to it.

**Table 5:** The result of the two-piecewise linear regression model

Outcome: incident stroke	HR (95%CI)	*P-*value
Fitting model by standard linear regression	1.049 (1.029, 1.069)	< 0.001
Inflection points of TyG-BMI	174.63	
< 174.63	1.144 (1.044, 1.253)	0.004
≥ 174.63	1.038 (1.016, 1.061)	< 0.001
*P* for log-likelihood ratio test	0.046	

Adjusted age, CRP, eGFR, sex; HDL-c, LDL-c, UA, CLD, PLT, Cystatin C; Hypertension, HBA1C, DM; CKD, smoking, drinking status

### Results of subgroup analysis

The link between TyG-BMI and stroke risk was not affected by sex, age, smoking status, hypertension, and drinking status in any of the prespecified or exploratory subgroups examined (Table 6). That is to say, the interaction between these variables and TyG-BMI was not statistically significant (P > 0.05 for interaction).

**Table 6:** Stratified associations between TyG-BMI and stroke by age, sex, hypertension, smoking status, and drinking status

Characteristics	No of participants	HR (95%CI)	*P* value	*P* for interaction
Age (years)				0.851
< 60	4731	1.064 (1.038, 1.091)	< 0.001	
60–70	2618	1.035 (1.006, 1.065)	0.017	
70–80	1141	1.024 (0.984, 1.065)	0.245	
≥ 80	208	1.009 (0.913, 1.115)	0.858	
Sex				0.767
Male	4008	1.052 (1.024, 1.081)	< 0.001	
Female	4690	1.047 (1.024, 1.071)	< 0.001	
Hypertension				0.054
No	6642	1.064 (1.039, 1.091)	< 0.001	
Yes	2056	1.032 (1.006, 1.059)	0.014	
Drinking status				0.135
Current drinker	1188	1.013 (0.974, 1.055)	0.512	
Ever drinker	2185	1.058 (1.035, 1.082)	< 0.001	
Never drinker	5325	1.048 (1.011, 1.085)	0.010	
Smoking status				0.951
Current smoker	2639	1.046 (1.014, 1.079)	0.005	
Ever smoker	745	1.038 (0.991, 1.087)	0.113	
Never smoker	5314	1.051 (1.027, 1.075)	< 0.001	

Above model adjusted for age, CRP, eGFR, sex; HDL-c, LDL-c, UA, CLD, PLT, Cystatin C; Hypertension, HBA1C, DM; CKD, smoking, drinking status. In each case, the model is not adjusted for the stratification variable

*HR* Hazard ratios, *CI* confidence, *Ref* reference

## Discussion

In this study, the connection between TyG-BMI and stroke incidence among middle-aged and senior individuals was evaluated. The findings revealed that a rise in TyG-BMI was associated with a significantly heightened risk of stroke. Additionally, an inflection point was identified, and different relationships between the TyG-BMI and stroke risk were detected on both sides.

The TyG index was first reported in 2008 and is considered a reliable, inexpensive, and simple surrogate for IR [37]. Many studies have confirmed that TyG is significantly associated with the incidence of atherosclerotic cardiovascular disease, including stroke [38–43]. A cohort study from the United States showed that after adjusting for potential confounders, each one-unit increase in the TyG index was associated with a 32.1% increase in the risk of stroke [43]. Another cohort study from China also showed a 34% increase in stroke risk for every 1-SD increase in the TyG index, with an adjusted HR of 1.34 (95% CI 1.11 to 1.61) [44]. In addition, the link between obesity and an elevated risk of stroke has been corroborated by numerous studies. BMI is frequently used as a measure of obesity, and a substantial positive correlation between BMI and the incidence of stroke has been documented in various research [34, 45, 46]. TyG-BMI is an obesity-related parameter that has been developed in recent years and has been strongly associated with NAFLD, cardiovascular events, prehypertension, and DM [21–23]. TyG-BMI is the product of BMI and TyG. This composite measure is believed to be a more accurate indicator of IR than individual indices. Given the known positive associations of both TyG and BMI with the risk of stroke and the critical role that IR is understood to play in stroke pathogenesis, we hypothesized that TyG-BMI might be positively correlated with stroke risk. Unfortunately, there is a scarcity of research investigating the connection between TyG-BMI and stroke risk, with only one study addressing this topic. A cross-sectional study from northeastern China showed that after multivariate adjustment, the risk of ischemic stroke increased by 20% for each standard deviation increase in TyG-BMI (OR = 1.20, 95% CI 1.10–1.32) [24]. Participants in the second and third tertiles of TyG-BMI had a significantly higher risk of ischemic stroke compared with those in the lowest tertile, with OR (95% CI) values of 1.39 (1.10–1.74) and 1.72 (1.37–2.17), respectively [24]. Our study expands on prior research supporting the hypothesis of a positive association between an elevated TyG-BMI index and stroke risk. Unlike previous studies, our investigation analyzed the TyG-BMI index as both categorical and continuous variables in relation to stroke risk, thus reducing information loss and quantifying their relationship. In addition, the K-means algorithm with Euclidean distance and logistic regression model were used to explore the association between changes in TyG-BMI and stroke risk, and it was found that those with consistently higher TyG-BMI had a similarly significant increase in stroke risk. Furthermore, sensitivity analyses specifically focused on participants who reported no CKD, no DM, and a BMI < 24 kg/m^2^. The results of the sensitivity analyses further confirmed that the relationships still existed in this group of participants. These results validate the stability of our findings. The identification of TyG-BMI as a risk factor for stroke and the elucidation of the relationship between the two provides a new perspective on stroke prevention and management, which is beneficial to patients' health outcomes and quality of life. In addition, it may lead clinicians and public health experts to revisit stroke risk assessment and prevention strategies.

The associations of TG, FPG, TyG, and BMI with stroke risk were further analyzed using multivariate Cox proportional hazards regression models. It was found that neither TG alone nor FPG was significantly associated with stroke risk, whereas the product of TG and FPG, the TyG index, was significantly and positively associated with stroke risk. Other possible reasons for this discrepancy include (1) the TyG index is considered a biomarker for metabolic syndrome [47]. Metabolic syndrome is an important factor in stroke risk and includes various metabolic abnormalities such as hypertension, abdominal obesity, and hyperlipidemia [48, 49]. Therefore, the TyG index provides a more comprehensive picture of the impact of these metabolic abnormalities on stroke risk than a single FPG or TG level. (2) Studies have confirmed that the TyG index is associated with IR, which is an independent risk factor for stroke [15]. IR may lead to a variety of pathologic changes, such as endothelial dysfunction, enhanced inflammatory response, and increased tendency to thrombosis, all of which may increase stroke risk [7, 15]. (3) FPG and TG can interact: Using FPG or TG levels alone to predict stroke risk may not be sensitive enough. However, if the two are combined into a single product, it may be possible to better reveal their interactions and synergistic effects in the metabolic process and thus more accurately predict stroke risk. In addition, BMI was also significantly and positively associated with stroke risk. Therefore, the tendency is to believe that the independent effect of TyG-BMI on stroke risk is based on the combined effect of TG, FPG, and BMI.

Although the precise mechanisms are not fully understood, the relationship between the TyG-BMI index and stroke risk is potentially linked to IR. The index is a composite measure that includes FPG, TG, and BMI, which serve as IR indicators. FPG levels reflect insulin sensitivity in the liver and insulin secretion in the pancreas [50]. In addition, the role of BMI and TG in recognizing IR has been demonstrated in previous studies [51–53]. TyG-BMI has been recommended as a marker for evaluating IR and IR-associated diseases [54, 55]. First, IR is implicated in the onset of atherosclerosis, fostering endothelial dysfunction, the emergence of foam cells, and the development of plaques prone to rupture [56–58]. Additionally, IR is often accompanied by a persistent state of mild inflammation, which can accelerate atherosclerotic processes and stimulate the release of pro-inflammatory markers [59, 60]. Moreover, IR can alter platelet function, leading to increased adhesion, activation, and aggregation, which may result in arterial narrowing or blockage, potentially leading to stroke [61, 62]. Therefore, the potential mechanism underlying the relationship between TyG-BMI and stroke incidence may be related to the association of three factors, FPG, TG, and BMI, with IR.

In addition, for the first time in our study, a non-linear relationship between TyG-BMI and stroke risk was observed. The inflection point of TyG-BMI was determined to be 174.63. When TyG-BMI was greater than 174.63, the risk of stroke decreased by 3.8% for every 10-unit decrease in TyG-BMI. On the other hand, when TyG-BMI was less than 174.63, the risk of stroke decreased by 14.4% for every 10-unit decrease in TyG-BMI. In other words, as the patient's TyG-BMI decreases, the risk of stroke gradually decreases. However, when TyG-BMI falls below 174.63, the stroke risk will fall more significantly. Further analysis showed that participants with TyG-BMI ≤ 174.63 had lower DBP, LDL-c, HB1AC, SBP, PLT, and UA, along with higher HDL-c, compared to those with TyG-BMI > 174.63. Additionally, those with TyG-BMI ≤ 174.63 had lower rates of CKD, DM, and hypertension (Additional file 1: Table S5). However, these indicators were strongly tied to stroke incidence [32–34, 63–67]. Due to these risk factors, the effect of TyG-BMI on stroke was relatively weak when TyG-BMI exceeded 174.63. In contrast, for those with TyG-BMI under 174.63, these stroke risk factors were lower and had less impact on stroke, and the role of TyG-BMI is relatively enhanced. This may explain the non-linear relationship between TyG-BMI and stroke risk. This finding of a curvilinear relationship between TyG-BMI and stroke has important clinical value. It facilitates clinical counseling and provides a basis for decision-making in stroke prevention. Combined reduction of BMI, TG, and FPG through dietary intervention and lifestyle changes can reduce the risk of stroke, and this risk will be significantly reduced by keeping TyG-BMI below 174.63. A previous cross-sectional study applied restricted cubic spline regression to analyze a possible non-linear relationship between TyG-BMI and ischemic stroke in the two cohorts included [24]. The results showed a linear relationship between TyG-BMI and ischemic stroke, with no threshold or saturation effect between the two. This is inconsistent with our findings of a non-linear relationship between TyG-BMI and stroke risk. The reasons may be as follows: first, the types of studies were different; the previous study was a cross-sectional study, whereas our study was a prospective cohort study. Second, the study populations were inconsistent; the previous study was a general population, whereas our study was a middle-aged and older population aged > 45 years. In addition, there were differences in the study methodology; the previous study used restricted cubic spline logistic regression to assess non-linear relationships. Whereas our study was a Cox proportional hazards model with cubic spline function. There were also differences in the covariates adjusted for, as we adjusted for more covariates, including UA, eGFR, DM, cystatin C, and CRP.

In the present study, several strengths have been identified. First, the application of both categorical and continuous TyG-BMI as independent variables was utilized to evaluate their association with stroke risk. This methodology was instrumental in diminishing information loss and in the precise quantification of the relationship under scrutiny. Second, the issue of missing data was addressed through the adoption of multiple imputation techniques. This strategy has been recognized for its capacity to enhance statistical power and for its role in minimizing bias that might arise from missing covariate data. Third, compared with previous studies, our study showed a significant improvement in the treatment of nonlinearity; for the first time, we found a non-linear relationship between TyG-BMI and stroke incidence. In addition, we performed a series of sensitivity analyses to ensure the stability of our findings. These analyses included exploring the relationship between changes in TyG-BMI (2011 to 2015) and stroke risk based on the K-means algorithm and multivariate logistic regression. The association between TyG-BMI and stroke incidence was reassessed after excluding subjects with DM, a BMI greater than 24 kg/m^2^, and CKD. Furthermore, the E-value was computed to assess the impact of potential unmeasured confounding factors, further affirming the study's findings.

The study in question presents certain limitations that warrant attention. Initially, the demographic focus on middle-aged and elderly individuals from China raises questions regarding the applicability of the findings to younger cohorts and different ethnic groups. To address this, future collaboration with international researchers is planned to explore these associations across varied genetic backgrounds. Additionally, the original dataset lacked certain stroke-related metrics, such as waist-to-hip ratio, medication usage, and familial stroke history. Third, as with all observational studies, residual confounding by unmeasured or uncontrolled confounders may remain despite adjustment for known potential confounders. However, we calculated E-values to assess the potential effect of unmeasured confounders, and the results suggested it was unlikely that these factors could entirely explain and influence our findings. Finally, this observational study could not ascertain causal relationships between TyG-BMI and stroke risk but only determine an association between them.

## Conclusion

The study found a significant link between higher TyG-BMI levels and an increased risk of stroke among middle-aged and elderly individuals in China. Notably, the relationship between TyG-BMI and stroke risk was non-linear. When the TyG-BMI value was below 174.63, any further decrease in TyG-BMI was associated with a marked reduction in the risk of stroke. This study provides additional references to facilitate clinical consultation and optimize stroke prevention decisions.

### Supplementary Information

**Additional file 1: Table S1.** Collinearity screening. **Table S2.** Factors influencing the risk of stroke were analyzed by univariate Cox proportional hazards regression. **Table S3.** Association of TG, FPG, TyG and BMI with stroke risk in different models. **Table S4.** Multivariate logistic regression analysis of the association between different TyG-BMI change groups (change from 2011 to 2015) and stroke risk. **Table S5.** The Baseline Characteristics of participants on both sides of the inflection point. **Figure S1.** showed the distribution of TyG-BMI across survey years after categorizing participants using the K-means algorithm. It was observed that participants in Class 1 exhibited overall low TyG-BMI levels (2011: 182.77 ± 22.56; 2015: 185.26 ± 24.08), whereas overall high TyG-BMI levels were exhibited by participants in Class 2 (2011: 245.52 ± 27.03; 2015: 250.24 ± 31.61).

## Data Availability

The data for this study can be accessed online at http://www.isss.pku.edu.cn/cfps/. To obtain the data, you will need to register as a user on the website. After your registration is reviewed and approved, you can follow the provided instructions to download the data set.

## Abbreviations

TyG-BMI: Triglyceride glucose-body mass index
HDL-c: High-density lipoprotein cholesterol
BMI: Body mass index
BUN: Blood urea nitrogen
LDL-c: Low-density lipoproteins cholesterol
FPG: Fasting plasma glucose
TG: Triglyceride
ALT: Alanine aminotransferase
SBP: Systolic blood pressure
TC: Total cholesterol
DBP: Diastolic blood pressure
GAM: Generalized additive models
Scr: Serum creatinine
OR: Odds ratio
eGFR: Estimated glomerular filtration rate
CKD: Chronic kidney diseases
UA: Uric acid
CLD: Chronic lung diseases
PLT: Platelet
DM: Diabetes mellitus
GAM: Generalized additive model
HBA1c: Hemoglobin
Ref: Reference
CI: Confidence interval
HR: Hazard ratio
