# Supplementary material for: Link between triglyceride-glucose-body mass index and future stroke risk in middle-aged and elderly chinese: a nationwide prospective cohort study
Source: Cardiovasc Diabetol. 2024 Feb 24;23:81. doi: 10.1186/s12933-024-02165-7 (PMC10893757; doi:10.1186/s12933-024-02165-7)
Supplement: Supplementary file 1 — Additional file 1: Table S1. Collinearity screening. Table S2. Factors influencing the risk of stroke were analyzed by univariate Cox proportional hazards regression. Table S3. Association of TG, FPG, TyG and BMI with stroke risk in different models. Table S4. Multivariate logistic regression analysis of the association between different TyG-BMI change groups (change from 2011 to 2015) and stroke risk. Table S5. The Baseline Characteristics of participants on both sides of the inflection point. Figure S1. showed the distribution of TyG-BMI across survey years after categorizing participants using the K-means algorithm. It was observed that participants in Class 1 exhibited overall low TyG-BMI levels (2011: 182.77 ± 22.56; 2015: 185.26 ± 24.08), whereas overall high TyG-BMI levels were exhibited by participants in Class 2 (2011: 245.52 ± 27.03; 2015: 250.24 ± 31.61). [file 12933_2024_2165_MOESM1_ESM.docx]

**Link Between Triglyceride-Glucose-Body Mass Index and Future Stroke Risk in Middle-Aged and Elderly Chinese: A nationwide prospective cohort study.**

**Running title:** TyG-BMI and stroke

Yuankai Shao^1#^, Haofei Hu^2#^, Qiming LI^1#^, Changchun Cao^3#^, Dehong Liu^1*^, Yong Han^1*^.

^1^Department of Emergency, Shenzhen Second People's Hospital, Shenzhen 518035, Guangdong Province, China.

^2^ Department of Nephrology, Shenzhen Second People's Hospital, Shenzhen 518035, Guangdong Province, China.

^3^ Department of Rehabilitation, Shenzhen Dapeng New District Nan'ao People's Hospital, Shenzhen 518000, Guangdong Province, China

Yuankai Shao, Haofei Hu, Qiming LI, and Changchun Cao have contributed equally to this work.

*Corresponding author

Dehong Liu

Department of Emergency, Shenzhen Second People's Hospital

No.3002 Sungang Road, Futian District,

Shenzhen 518035,

Guangdong Province,

China

E-mail: dhliu_emergency@163.com

*Corresponding author

Yong Han

Department of Emergency, Shenzhen Second People's Hospital

No.3002 Sungang Road, Futian District,

Shenzhen 518035,

Guangdong Province,

China.

Hanyong511023@163.com

Table S1 Collinearity screening

|  | Step 1 | Step 2 | Step 3 | Step 4 |
| --- | --- | --- | --- | --- |
| TyG-BMI | 3.1 | 3.1 | 3.1 | 2.7 |
| Age | 1.6 | 1.5 | 1.5 | 1.5 |
| Sex | 2.5 | 2.5 | 2.5 | 2.4 |
| WBC | 1.2 | 1.2 | 1.2 | 1.2 |
| PLT | 1.1 | 1.1 | 1.1 | 1.1 |
| BUN | 1.2 | 1.2 | 1.2 | 1.2 |
| Scr | 2.1 | 2.1 | 2.1 | 2.1 |
| TC | 11.3 | 11.3 | NA | NA |
| HDL-c | 3.1 | 3.1 | 1.5 | 1.5 |
| LDL-c | 8.6 | 8.5 | 1.1 | 1.1 |
| CPR | 1.1 | 1.1 | 1.1 | 1.1 |
| HBA1C | 1.9 | 1.9 | 1.9 | 1.9 |
| UA | 1.5 | 1.5 | 1.5 | 1.5 |
| Cystatin C | 2.1 | 2.1 | 2 | 2 |
| SBP | 2.7 | 2.7 | 2.7 | 2.7 |
| DBP | 2.5 | 2.5 | 2.5 | 2.5 |
| Hypertension | 1.3 | 1.3 | 1.3 | 1.3 |
| DM | 1.2 | 1.2 | 1.2 | 1.2 |
| CLD | 1.1 | 1.1 | 1.1 | 1 |
| CKD | 1 | 1 | 1 | 1 |
| Smoking | 1.9 | 1.9 | 1.9 | 1.9 |
| Drinking | 1.1 | 1.1 | 1.1 | 1.1 |
| eGFR | 4.2 | 4.2 | 4.2 | 4.2 |

Variables excluded from collinearity screening: TC

Abbreviations: LDL-c, low-density lipoproteins cholesterol; BUN, blood urea nitrogen; TyG-BMI, Triglyceride glucose-body mass index; WBC, white blood cell count ; PLT, platelet; BMI, body mass index; HGB, hemoglobin concentration; UA, Uric acid; TC, total cholesterol; HBA1c, hemoglobin A1c; DBP, diastolic blood pressure; TG, triglyceride; eGFR, Estimated glomerular filtration rate; CLD, Chronic Lung Diseases; DM, diabetes mellitus; Scr, serum creatinine; HDL-c, high-density lipoprotein cholesterol; CKD, Chronic kidney diseases; SBP, systolic blood pressure.

Table S2. Factors influencing the risk of stroke were analyzed by univariate Cox proportional hazards regression.

|  | Subject characteristics | (HR.,95%CI) *p* |
| --- | --- | --- |
| Age (years, mean ± SD) | 59.4 ± 9.3 | 1.031 (1.025, 1.038) <0.001 |
| Sex |  |  |
| Male | 4008 (46.08%) | Ref |
| Female | 4690 (53.92%) | 1.147 (1.012, 1.301) 0.032 |
| WBC (10^9/L, mean ± SD) | 6.24 ± 1.89 | 1.040 (1.008, 1.074) 0.014 |
| PLT (10^9/L, ,mean ± SD) | 211 ± 73 | 1.001 (1.000, 1.002) 0.019 |
| BUN (mg/L, mean ± SD) | 43.96 ± 12.88 | 1.002 (0.988, 1.016) 0.749 |
| FPG (mg/L, mean ± SD) | 109.98 ± 36.36 | 1.003 (1.002, 1.004) <0.001 |
| Scr(mg/dL, mean ± SD) | 0.78 ± 0.24 | 1.344 (1.123, 1.608) 0.001 |
| TC (mg/dL, mean ± SD) | 193.61 ± 38.36 | 1.002 (1.001, 1.004) 0.002 |
| TG (mg/dL, median, quartile) | 105.32 (74.34-153.10) | 1.001 (1.000, 1.001) 0.001 |
| TyG-BMI (mean ± SD) | 20.351 ± 3.866 | 1.071 (1.055, 1.087) <0.001 |
| eGFR(mL/min·1.73 m^2^, mean ± SD) | 96.85 ± 15.58 | 0.985 (0.982, 0.989) <0.001 |
| TyG (mean ± SD) | 8.68 ± 0.66 | 1.341 (1.231, 1.461) <0.001 |
| HDL-c(mg/dL, mean ± SD) | 51.45 ± 15.29 | 0.991 (0.987, 0.995) 0.004 |
| LDL-c(mg/dL, mean ± SD) | 116.67 ± 34.92 | 1.002 (1.001, 1.004) 0.009 |
| CRP (mg/L, median, quartile) | 1.02 (0.55-2.13) | 1.008 (1.002, 1.015) 0.008 |
| HBA1C (%, mean ± SD) | 5.27 ± 0.80 | 1.205 (1.137, 1.278) <0.001 |
| UA (mg/dL, mean ± SD) | 4.44 ± 1.24 | 1.073 (1.022, 1.127) 0.005 |
| HGB(g/dL, mean ± SD) | 14.30 ± 2.22 | 1.013 (0.986, 1.042) 0.353 |
| Cystatin C (mg/L, mean ± SD) | 1.01 ± 0.27 | 1.524 (1.290, 1.800) <0.001 |
| SBP (mmHg, mean ± SD) | 129 ± 22 | 1.014 (1.011, 1.016) <0.001 |
| DBP (mmH, mean ± SD) | 75 ± 12 | 1.018 (1.013, 1.023) <0.001 |
| BMI (kg/m^2^, mean ± SD) | 23.4 ± 3.6 | 1.066 (1.049, 1.084) <0.001 |
| Hypertension(n, %) |  |  |
| No | 6642 (76.362%) | Ref |
| Yes | 2056 (23.638%) | 2.638 (2.327, 2.989) <0.001 |
| DM(n, %) |  |  |
| No | 8224 (94.550%) | Ref |
| Yes | 474 (5.450%) | 2.373 (1.946, 2.893) <0.001 |
| CLD(n, %) |  |  |
| No | 7781 (89.457%) | Ref |
| Yes | 917 (10.543%) | 1.744 (1.472, 2.066) <0.001 |
| CKD(n, %) |  |  |
| No | 8120 (93.355%) | Ref |
| Yes | 578 (6.645%) | 1.565 (1.269, 1.929) <0.001 |
| Smoking status(n, %) |  |  |
| Never | 5325 (61.221%) | Ref |
| Ever | 741 (8.519%) | 1.467 (1.201, 1.792) <0.001 |
| Current | 2632 (30.260%) | 0.956 (0.830, 1.101) 0.5351 |
| Drinking status(n, %) |  |  |
| Never | 5325 (61.221%) | Ref |
| ever | 2185 (25.121%) | 0.853 (0.731, 0.995) 0.043 |
| current | 1188 (13.658%) | 1.231 (1.037, 1.461) 0.018 |

Abbreviations: SD, standard deviation; N, number; LDL-c, low-density lipoproteins cholesterol; BUN, blood urea nitrogen; TyG-BMI, Triglyceride glucose-body mass index; WBC, white blood cell count ; PLT, platelet; BMI, body mass index; HGB, hemoglobin concentration; UA, Uric acid; TC, total cholesterol; HBA1c, hemoglobin A1c; DBP, diastolic blood pressure; TG, triglyceride; eGFR, Estimated glomerular filtration rate; CLD, Chronic Lung Diseases; DM, diabetes mellitus; Scr, serum creatinine; HDL-c, high-density lipoprotein cholesterol; CKD, Chronic kidney diseases; SBP, systolic blood pressure.

HR, hazard ratio; Ref: reference; CI: confidence.

**Table S3 Association of TG, FPG, TyG and BMI with stroke risk in different models.**

|  | Model | (HR.,95%CI) *p* |
| --- | --- | --- |
| Metabolic or electrolyte abnormality | a | 1.024 (0.970, 1.080) 0.389 |
| FPG(mmol/L) | b | 1.015 (0.987, 1.044) 0.287 |
| TyG | c | 1.165 (1.043, 1.302) 0.007 |
| BMI(kg/m^2^) | d | 1.045 (1.025, 1.064) <0.001 |

Model a: we adjusted age, BMI, FPG, CRP, eGFR, sex, HDL-c, LDL-c, UA, CLD, PLT, Cystatin C, hypertension, HBA1C, DM, CKD, smoking, drinking status.

Model b: we adjusted age, BMI, TG, CRP, eGFR, sex, HDL-c, LDL-c, UA, CLD, PLT, Cystatin C, hypertension, HBA1C, DM, CKD, smoking, drinking status.

Model c: we adjusted age, BMI, CRP, eGFR, sex, HDL-c, LDL-c, UA, CLD, PLT, Cystatin C, hypertension, HBA1C, DM, CKD, smoking, drinking status.

Model d: we adjusted age, TG, FPG, CRP, eGFR, sex, HDL-c, LDL-c, UA, CLD, PLT, Cystatin C, hypertension, HBA1C, DM, CKD, smoking, drinking status.

HR, hazard ratio; CI: confidence.

**Table S4 Multivariate logistic regression analysis of the association between different TyG-BMI change groups (change from 2011 to 2015) and stroke risk**

|  | Model I(OR,95%CI) p | Model I(OR,95%CI) p | Model I(OR,95%CI) p |
| --- | --- | --- | --- |
| Class 1 | Ref | Ref | Ref |
| Class 2 | 1.398 (1.170, 1.670) <0.001 | 1.468 (1.223, 1.763) <0.001 | 1.241 (1.012, 1.523) <0.001 |

Model I: we did not adjust other covariates.

Model II: we adjust sex and age.

Model III: we adjust age, CRP, eGFR, sex, HDL-c, LDL-c, UA, CLD, PLT, Cystatin C, HBA1C, DM, CKD, smoking, drinking status.

OR, odds ratio; Ref: reference; CI: confidence.

**Table S5 The Baseline Characteristics of participants on both sides of the inflection point**

| TyG-BMI groups | <174.63 | >=174.63 | P-value |
| --- | --- | --- | --- |
| Participants(n) | 2127 | 6571 |  |
| Age (years, mean ± SD) | 61.97 ± 9.89 | 58.51 ± 8.89 | <0.001 |
| PLT (10^9/L, mean ± SD) | 208.75 ± 76.58 | 212.40 ± 71.57 | 0.044 |
| BUN (mg/L, mean ± SD) | 45.65 ± 14.07 | 43.55 ± 12.27 | <0.001 |
| FPG (mg/L , mean ± SD) | 99.17 ± 18.72 | 113.48 ± 39.83 | <0.001 |
| Scr (mg/L , mean ± SD) | 0.78 ± 0.24 | 0.78 ± 0.23 | 0.395 |
| TC (mg/dL, mean ± SD) | 183.79 ± 35.23 | 196.78 ± 38.79 | <0.001 |
| TG (mg/dL, median, quartile) | 72.57 (57.53-94.69) | 120.36 (85.85-172.57) | <0.001 |
| TyG(mean ± SD) | 8.19 ± 0.44 | 8.84 ± 0.65 | <0.001 |
| HDL-c (mg/dL, mean ± SD) | 59.97 ± 15.82 | 48.70 ± 14.05 | <0.001 |
| eGFR(mL/min·1.73 m^2^, mean ± SD) | 96.52 ± 15.50 | 96.95 ± 15.61 | 0.268 |
| LDL-c(mg/dL, , mean ± SD) | 109.72 ± 30.67 | 118.92 ± 35.90 | <0.001 |
| CRP (mg/L, median, quartile) | 0.77 (0.44-1.84) | 1.09 (0.59-2.19) | 0.121 |
| HBA1C (%,, mean ± SD) | 5.10 ± 0.52 | 5.32 ± 0.87 | <0.001 |
| UA (mg/dL , mean ± SD) | 4.29 ± 1.21 | 4.49 ± 1.25 | <0.001 |
| HGB(g/dL, , mean ± SD) | 13.97 ± 2.22 | 14.41 ± 2.21 | <0.001 |
| Cystatin C (mg/L, mean ± SD) | 1.07 ± 0.27 | 0.99 ± 0.27 | <0.001 |
| SBP (mmHg , mean ± SD) | 124.81 ± 21.85 | 130.63 ± 21.29 | <0.001 |
| DBP (mmHg , mean ± SD) | 71.79 ± 11.79 | 76.67 ± 12.14 | <0.001 |
| BMI (kg/m^2^ , mean ± SD) | 19.32 ± 1.69 | 24.69 ± 3.01 | <0.001 |
| Sex(n, %) |  |  | <0.001 |
| Male | 1218 (57.26%) | 2790 (42.46%) |  |
| Female | 909 (42.74%) | 3781 (57.54%) |  |
| CKD (n, %) | 165 (7.76%) | 413 (6.29%) | 0.018 |
| Smoking status(n, %) |  |  | <0.001 |
| Never | 1058 (49.74%) | 4267 (64.94%) |  |
| Ever | 169 (7.95%) | 572 (8.70%) |  |
| Current | 900 (42.31%) | 1732 (26.36%) |  |
| Hypertension(n.%) | 262 (12.32%) | 1794 (27.30%) | <0.001 |
| CLD(n, %) | 311 (14.62%) | 606 (9.22%) | <0.001 |
| DM(n, %) | 36 (1.69%) | 438 (6.67%) | <0.001 |
| Drinking status(n, %) |  |  | <0.001 |
| Never | 1200 (56.42%) | 4125 (62.78%) |  |
| Ever | 302 (14.20%) | 886 (13.48%) |  |
| Current | 625 (29.38%) | 1560 (23.74%) |  |

Abbreviations: SD, standard deviation; N, number; LDL-c, low-density lipoproteins cholesterol; BUN, blood urea nitrogen; TyG-BMI, Triglyceride glucose-body mass index; PLT, platelet; BMI, body mass index; HGB, hemoglobin concentration; UA, Uric acid; TC, total cholesterol; HBA1c, hemoglobin A1c; DBP, diastolic blood pressure; TG, triglyceride; eGFR, Estimated glomerular filtration rate; CLD, Chronic Lung Diseases; DM, diabetes mellitus; Scr, serum creatinine; HDL-c, high-density lipoprotein cholesterol; CKD, Chronic kidney diseases; SBP, systolic blood pressure.


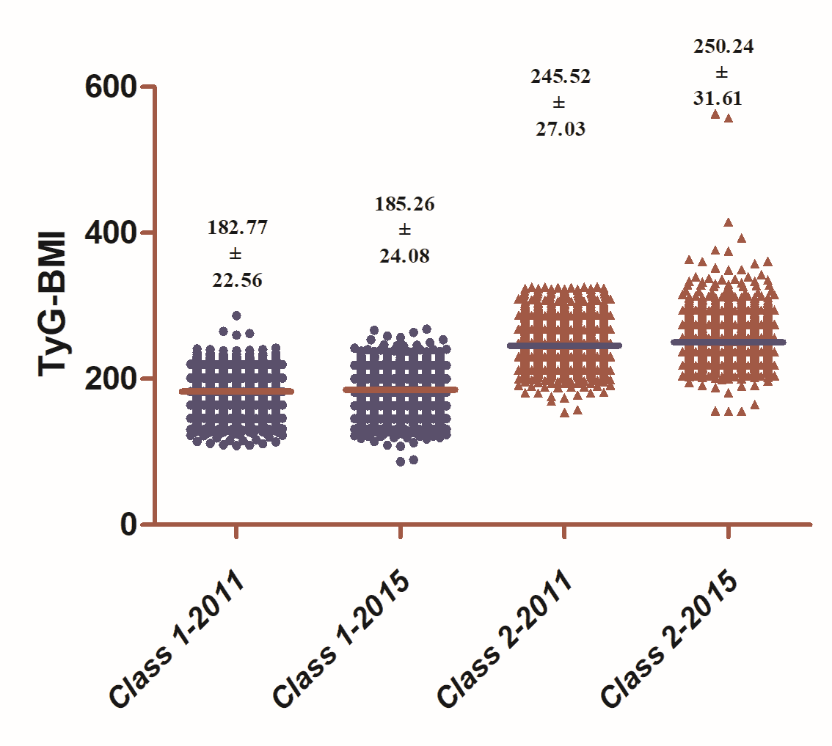


**Figure S1** showed the distribution of TyG-BMI across survey years after categorizing participants using the K-means algorithm. It was observed that participants in Class 1 exhibited overall low TyG-BMI levels (2011: 182.77 ± 22.56; 2015: 185.26 ± 24.08), whereas overall high TyG-BMI levels were exhibited by participants in Class 2 (2011: 245.52 ± 27.03; 2015: 250.24 ± 31.61).
